# Adult-Onset Still’s Disease: Clinical Aspects and Therapeutic Approach

**DOI:** 10.3390/jcm10040733

**Published:** 2021-02-12

**Authors:** Stylianos Tomaras, Carl Christoph Goetzke, Tilmann Kallinich, Eugen Feist

**Affiliations:** 1 Department of Rheumatology, Helios Clinic Vogelsang-Gommern, 39245 Gommern, Germany; Eugen.Feist@helios-gesundheit.de; 2 Department of Pediatrics, Division of Pulmonology, Immunology and Critical Care Medicine, Charité–Universitätsmedizin Berlin, Corporate Member of Freie Universität Berlin, Humboldt-Universität zu Berlin, and Berlin Institute of Health (BIH), 10117 Berlin, Germany; carl-christoph.goetzke@charite.de (C.C.G.); tilmann.kallinich@charite.de (T.K.); 3 German Rheumatism Research Center (DRFZ), Leibniz Association, 10117 Berlin, Germany; 4 Berlin Institute of Health, 10178 Berlin, Germany

**Keywords:** adult-onset Still’s disease, autoinflammatory disorder, systemic-onset juvenile idiopathic arthritis, haemophagocytic lymphohistiocytosis, macrophage activation syndrome

## Abstract

Adult-onset Still’s disease (AoSD) is a rare systemic autoinflammatory disease characterized by arthritis, spiking fever, skin rash and elevated ferritin levels. The reason behind the nomenclature of this condition is that AoSD shares certain symptoms with Still’s disease in children, currently named systemic-onset juvenile idiopathic arthritis. Immune dysregulation plays a central role in AoSD and is characterized by pathogenic involvement of both arms of the immune system. Furthermore, the past two decades have seen a large body of immunological research on cytokines, which has attributed to both a better understanding of AoSD and revolutionary advances in treatment. Additionally, recent studies have introduced a new approach by grouping patients with AoSD into only two phenotypes: one with predominantly systemic features and one with a chronic articular disease course. Diagnosis presupposes an extensive diagnostic workup to rule out infections and malignancies. The severe end of the spectrum of this disease is secondary haemophagocytic lymphohistiocytosis, better known as macrophage activation syndrome. In this review, we discuss current research conducted on the pathogenesis, diagnosis, classification, biomarkers and complications of AoSD, as well as the treatment strategy at each stage of the disease course. We also highlight the similarities and differences between AoSD and systemic-onset juvenile idiopathic arthritis. There is a considerable need for large multicentric prospective trials.

## 1. Introduction

Adult-onset Still’s disease (AoSD) is a rare systemic autoinflammatory disease characterized by arthritis, spiking fever, skin rash and elevated ferritin levels. The cause of this complex disorder, which usually affects young adults, remains unknown [1]. A London doctor named Bywaters first introduced the term AoSD in the medical literature in 1971 by describing this condition in a small group of 14 patients with an age range of 17 to 35 years [2]. The reason behind the nomenclature of this disease is that AoSD shares certain symptoms with Still’s disease in children, which is currently named systemic-onset juvenile idiopathic arthritis (SoJIA). Based on gene expression analysis, some regard SoJIA and AoSD as a single nosological entity [3]. Most recent estimates place AoSD incidence at 0.16 to 0.4 per 100,000 persons [4].

One of the most interesting current discussions in immunology is the newly introduced concept of a “crossroads between autoinflammation and autoimmunity due to the pathogenic involvement of both arms of the immune system” [5]. AoSD, like PFAPA (periodic fever with aphthous stomatitis, pharyngitis and adenitis) and Behçet’s disease, is a complex disorder with malfunctioning dysregulated immune system. On the one hand, it lacks the classical characteristics of autoimmune diseases, such as autoantibodies, but on the other hand, it has negative genetic testing in family histories, which is opposite to other autoinflammatory conditions [6].

The past two decades have seen a large body of immunological research on cytokines, which has attributed to both a better understanding of AoSD and significant advances in treatment. One major problem is that although biological drugs have made revolutionary changes in the management of a range of rheumatic conditions, many patients with AoSD are not benefiting from most of them [7]. In addition, every rheumatologist with a patient who had a life-threatening cytokine storm during macrophage activation syndrome (MAS) has deep respect for AoSD. 

The goal of our paper is to summarize the current (2020) state of knowledge on the pathogenesis, diagnosis, classification, biomarkers and complications of AoSD, as well as the treatment strategy at each stage of the disease course.

## 2. Autoinflammation and Autoimmunity

Autoimmunity was, historically, defined as a dysregulation of the adaptive immune system, exclusively involving B and T lymphocytes and leading to the production of autoantibodies directed against self. Autoinflammation, on the other hand, was strictly separated from autoimmunity and was previously considered to have a solely innate autoimmune aetiology. Recent studies on pattern recognition receptors (PRRs) were the breakthrough discovery that changed the way we approach these two phenomena and elucidated the pathology of a group of disorders where both arms interfere and contribute to the inflammatory response [8]. 

Autoinflammation in periodic fever syndromes is caused by an inborn error of the innate immune system that results in the perturbation of pattern recognition receptors (PRRs), such as the leucine-rich repeat containing family (NLR), leading to an inappropriate chain reaction towards both pathogen-associated molecular patterns (PAMPs) and damage-associated molecular patterns molecules released from injured tissues (DAMPs) [9].

In concert with this theory, genetic errors in the NLR pathway can trigger the onset of Crohn’s disease, a very well-known disorder that was classified as an autoimmune disease until recently. Currently, Crohn’s disease is considered an autoimmune disease with a prevalent autoinflammatory pathogenesis [10]. Moreover, a small subgroup of patients with rheumatoid arthritis show systemic inflammatory symptoms, such as fever and serositis, although this disease is not supposed to have a coexistent autoinflammatory background [11]. 

AoSD belongs to this group of disorders and is thought to be “the archetype of non-familial, or sporadic, systemic autoinflammatory disorders” [12].

### 2.1. Pathogenesis Part I: Who Started the Fire

The exact underlying cause of AoSD is not fully understood. We still do not know what exactly triggers DAMPs and PAMPs. 

The causal inferences between genetics and AoSD are controversial. Human genetic factors apparently contribute to SoJIA in children, whereas the underlying genomic susceptibility in the adult form is unclear [13].

On the other hand, there is a high degree of similarity between infections and the onset of AoSD for fever, leucocytosis and elevated C-reactive protein (CRP). Logically, many investigators focused on identifying infectious triggers and described the occurrence of AoSD after infection with cytomegalovirus, Epstein-Barr, influenza, *Mycoplasma*, hepatitis, etc. [4]. We now know that cytomegalovirus may also trigger a relapse of AoSD [14]. Blood cultures and polymerase chain reaction (PCR) tests may, therefore, be useful for a differential diagnosis, although no specific diagnostic algorithms exist to date. It is currently still not clear which pathogenic viruses and bacteria should be included in the diagnostic workup. Remarkably, procalcitonin is not a reliable marker, since patients suffering from AoSD can show elevated procalcitonin levels without confirmed infection [15]. 

Other studies have examined the relationship between cancer and AoSD [16] and reported malignancy-mediated autoinflammation in breast cancer [17], thyroid cancer [18], melanoma, lung cancer and haematological malignancies, mostly lymphomas [19]. Despite increasing sophistication in the diagnostic workup for possible malignancies, there are no universally accepted guidelines for patients with AoSD, which makes daily clinical work more difficult. Positron emission tomography and computed tomography (PET/CT) scanning could be useful in difficult case scenarios to rule out solid tumours or large vessel vasculitis mimicking AoSD, but it is not routine practice because of the relatively high costs [20]. Bone marrow examination can rule out a haematologic malignancy or support the diagnosis of MAS. 

In short, AoSD is a diagnosis of exclusion. The process of eliminating similar medical conditions is most likely to take a considerable amount of time. Table 1 summarizes the broad spectrum of differential diagnoses.

### 2.2. Pathogenesis Part II: What Keeps the Fire Burning

PAMPs and DAMPs stimulate macrophages and neutrophils, leading to activation of specific inflammasomes via Toll-like receptors. Inflammasomes are multiprotein units that act as catalysts by activating the caspase pathway immediately after they come into contact with damage or illness. Caspase enzymes lead to overproduction of IL-1β, the hallmark of AoSD, and IL-18. IL-1β and IL-18 then promote further abnormal inflammation by several cytokine bursts, including IL-6, IL-8, IL-17, IL-18 and TNF-α. At this point, the patient is experiencing heavy systemic symptoms [21,22,23,24].

Furthermore, activated macrophages stimulate the release of excessive levels of ferritin. In addition to functioning as an iron storage molecule, ferritin also plays a central role in many conditions with an amplified inflammatory response, currently called “hyperferritinemic syndromes”, such as AoSD, MAS, catastrophic antiphospholipid syndrome and septic shock [25]. Ferritin has a key role in inflammation by promoting cytokine production, and at the same time, cytokines can regulate ferritin synthesis.

Moreover, analysis of accumulating data over the past years showed an enhancement of neutrophil extracellular traps (NET) in AoSD, which promotes the acute phase response by activating the NLRP3 inflammasome [26].

Additionally, dysfunctional natural killer (NK) cells, elevated T-helper Th1 and Th17 cells, enhanced IFN-γ and IL-17 levels, different alarmins, such as the S100 proteins, significantly higher IFN-γ-producing Th1 cells and Th1/Th2 cells ratios and advanced glycation end products complete the proinflammatory environment in many ways, which favours the abnormal response of the human immune system [27,28,29].

### 2.3. Pathogenesis Part III: Why Is Firefighting so Hard

The massive release of cytokines in patients with AoSD over a prolonged period of time can be fatal. Deficient resolution of inflammation may be mostly due to failures in immune system self-regulation. Deficient regulatory T cells, decreased or defective NK cells, insufficient production of anti-inflammatory cytokines or problematic circulation of advanced glycation end products (AGEs) have been hypothesized to cause these complex problems [30,31,32,33]. Surprisingly, the anti-inflammatory cytokine IL-10 levels are elevated during the higher state of inflammation and correlate with disease activity in AoSD [34]. 

## 3. Clinical Symptoms

Nonspecific symptoms such as fever, sore throat or arthralgia that usually bring patients with AoSD to medical attention are rather misleading. The similarities with an infection often obscure the diagnosis and lead to empirical antibiotic therapies. Italian and French studies have shown a diagnostic delay ranging from 1.5 to 4 years between the onset of symptoms and the final diagnosis of AoSD [4,35]. When all conservative treatments fail, practitioners realize they are facing a prolonged febrile illness without an obvious aetiology. The diagnostic journey then begins.

In a large retrospective study, which set out to analyse 1641 patients with fever of unknown origin (FUO), AoSD was responsible for 5.4% of cases [36]. Overall, rheumatic diseases comprise approximately 30% of cases with FUO, with AoSD being the most frequent group [37].

Fever is a cardinal symptom in AoSD and occurs in 60 to 100% of cases. Patients typically report two fever spikes daily, one in the morning and one in the evening, usually >39 °C. In 60 to 80% of patients, a macular or maculopapular evanescent salmon-pink skin rash on the proximal limbs and trunk accompanies high fever. Interestingly, this rash can disappear completely during afebrile intervals. Permanent skin rashes, on the other hand, presenting with urticaria, are warning signs for haematological complications. Both fever and skin rash are correlated with disease activity. Along with other nonspecific constitutional symptoms, such as weight loss and malaise, patients with active AoSD feel sick and miserable [1,4,38].

Arthralgia is also a cardinal symptom that is observed in 70 to 100% of patients, often accompanied by polyarthritis involving small joints, imitating rheumatoid arthritis. Some patients with chronic articular AoSD show severe osteodestructive features, which cause ankyloses and functional disability [39]. 

Other concomitant symptoms, such as pharyngitis, odynophagia, lymphadenopathy, splenomegaly, myalgia, pleuritis or abdominal pain vary from person to person. National registries and patient cohorts are a major determinant for successful characterization of clinical phenotypes in the field of rare diseases, such as AoSD. Table 2 shows the summary statistics of some observational studies and illustrates the heterogeneity of AoSD and SoJIA.

## 4. Laboratory Findings and Biomarkers

There are no pathognomonic laboratory findings in AoSD. Negative acute phase proteins allow exclusion of an active AoSD. Laboratory tests will almost always detect high levels of both CRP and leukocytes (>10,000/mm^3^), yet highly elevated leukocyte counts of >50,000/mm^3^ are usually associated with haematological malignancies. In contrast, leukopenia is related to an unfortunate course of disease with complications such as reactive haemophagocytic lymphohistiocytosis or thrombotic angiopathy.

Diagnostic workup should also include liver function tests, as nearly 50% of the patients show elevated transaminases, mostly due to non-steroidal anti-inflammatory drugs (NSAIDs) or antibiotics and rarely due to fulminant hepatitis [35]. 

Moreover, ferritin is a very helpful serologic marker for diagnosis and follow-up, especially when it increases >5-fold. Current propositions for hyperferritinaemia in AoSD include increased production by macrophages, liver and erythrocytes due to parallel erythrophagocytosis [46,47,48,49]. Furthermore, high circulating ferritin has a positive feedback mechanism that can further exacerbate its own inflammatory properties [24]. Ferritin contains two types of subunits: heavy (H) and light (L). In the bone marrow of patients with MAS, high levels of H-ferritin are found, and they correlate with disease severity. Correspondingly, lymph nodes and skin are infiltrated with CD68/H-ferritin cells. 

Several studies have pointed out the diagnostic utility of glycosylated ferritin (GF). A low percentage of GF is significantly related to amplifying inflammation in AoSD. A combined laboratory approach of GF <20% with ferritin levels >5-fold can optimize the diagnosis and yield a sensitivity of 43.2% and specificity of 92.9% [47]. Low GF can also be used as a biomarker for haemophagocytosis [50]. Remarkably, GF does not perform well in the assessment of disease activity in AoSD, since it remains low for several weeks or months after flare up [51]. Unfortunately, measurement of GF is not a common marker in routine laboratory diagnostics so far and few studies have been published supporting its relevance.

Serum cytokine levels, such as IL-1, IL-6 or IL-18, could be helpful to diagnose AoSD, but they are not yet recommended for routine practice [52,53,54].

Furthermore, studies investigating the members of the S100 protein family and how they interact with proinflammatory signalling pathways show that they could be a potential biomarker. However, more studies are needed to consider them a routine test [55,56]. 

High levels of serum amyloid A can predict the development of systemic amyloidosis [53].

## 5. Diagnostic Criteria

During the diagnostic process, most physicians use the Yamaguchi and Fautrel classification criteria for AoSD in actual practice, although they are primarily designed to select patients for clinical trials (Table 3).

One major limitation of the Yamaguchi criteria set is its exclusion criteria. This approach is not beneficial in clinical practice, as it presupposes an extensive diagnostic workup, whereas the needed laboratory and imaging tests are not specified. Another problem is that helpful biomarkers such as ferritin are not included. In contrast, Fautrel’s criteria provide a core set without exclusion criteria and refer to the usability of glycosylated ferritin as a diagnostic marker.

To validate the performance of the Fautrel criteria in 2018 in a different cohort than the original in 2002, a French working group included 54 patients with AoSD and 278 controls. The sensitivity was 87.0%, the specificity was 97.8%, and the positive and negative predictive values were 88.7% and 97.5%, respectively. In the same study, the Yamaguchi criteria (without exclusion restrictions) performed better and showed a sensitivity of 96.3% and a specificity 98.9%, with positive and negative predictive values of 94.5% and 99.3%, respectively [59]. 

## 6. The Course of the Disease Splits in Two

Several disease patterns have been observed in patient cohort studies. For approximately 19–44% of affected patients, AoSD has a monocyclic course without relapses. A polycyclic course is identified in 10–41% of affected patients and is characterized by unpredictable periods of exacerbation after a few months or years. Approximately 35–57% of affected patients show a chronic progressive course, which is the most frequent one, which is characterized by steady progression, continuous inflammation and often erosive joint involvement [60]. 

However, recent studies have introduced a new approach by grouping patients with AoSD into only two phenotypes: one with predominantly systemic features and one with a chronic articular disease course. Treatment of the systemic form is different from the treatment used for adults with progressive joint involvement, due to a higher inflammatory status and possible multi-organ damage with haematological complications. The non-systemic subgroup, on the other hand, may begin with systemic symptoms and evolve to a disease resembling rheumatoid arthritis at the end stage. This phenotypic dichotomy may also simplify the design of future clinical trials [4,61,62,63,64].

Predictive factors for the systemic subset of AoSD include high fever (>39 °C) and high levels of liver enzymes or CRP, while female sex, polyarthritis at disease onset and steroid dependence are associated with the chronic articular subgroup [65,66]. To close, this simplified theory of dichotomous disease courses is supported, at least partially, by studies on cytokine profiles and responses to biologic treatments [65,67,68].

## 7. Complications

### 7.1. Cytokine Storm

The most severe complication of the spectrum of Still’s disease and AoSD is secondary haemophagocytic lymphohistiocytosis (HLH), better known as MAS. The term cytokine storm best describes excessive cytokinaemia during MAS. The prevalence varies from 10 to 15% and is associated with high mortality [64]. Possible triggers such as infections or medications in combination with uncontrolled and prolonged inflammation in patients with genetic predisposition may lead to this life-threatening condition [69,70,71].

Researchers from France developed diagnostic criteria for MAS to shorten the critical process of reaching an accurate diagnosis. In this multicentre retrospective cohort study of 312 patients, the diagnosis relied on a set of nine variables: known underlying immunosuppression, high temperature, organomegaly, triglyceride, ferritin, serum aspartate transaminase, fibrinogen levels, cytopenia and haemophagocytosis features on bone marrow aspirate (Table 4). Based on a scoring system, physicians can then calculate the “HScore” and assess the probability of the patient having MAS. MAS can be ruled out with an HScore of ≤90 MAS, whereas an HScore ≥ 250 has a diagnostic accuracy of >99% [72]. 

Knowing how to diagnose MAS could be life-saving because of its short therapeutic window of opportunity. Even if the full diagnostic criteria are not met, treatment should be started as soon as possible to silence the cytokine storm and prevent hyperinflammatory complications, critical illness and death. Cross-specialty collaboration is the key to success. 

Once a diagnosis of MAS has been made, serum ferritin concentrations are useful for monitoring disease activity and response to treatment. Very high peak levels as well as a limited decrease (less than 50% from first measurement near diagnosis) after initiation of treatment are associated with high mortality in paediatric patients [73].

### 7.2. Parenchymal Lung Disease and PAH

The latest research in paediatrics reported lung involvement in children with SoJIA, a rare but potentially fatal complication [74,75]. Correspondingly, 12% of the 147 adult patients with AoSD included in Gruppo Italiano di Ricerca in Reumatologia Clinica e Sperimentale (GIRRCS) cohort have been diagnosed with parenchymal lung disease. Older age and higher inflammation status were independent predictors. Overall, the survival rate was significantly decreased in this subgroup [76]. The reason behind the high mortality rate is the association with MAS. Lung involvement seems to trigger accelerating mechanisms of inflammation. This observation reflects the data about the occurrence of MAS in children with lung damage [77,78]. Bronchiolitis and nonspecific interstitial pneumonia are the most common histological patterns [79]. Pulmonary hypertension in AoSD is a rare complication but it represents a life-threatening condition with a mortality of about 40%. This disorder mostly affects women and leads to rapidly progressive respiratory distress [80,81]. 

### 7.3. Coagulation Disorders

Disseminated intravascular coagulation (DIC) is a rare complication in patients, mainly in those with the systemic phenotype of AoSD and it occurs in 1–5% of cases. Cutaneous or mucosal bleeding and/or signs of thromboembolism are suggestive of DIC [82]. The DIC-Score by the International Society on Thrombosis and Haemostasis (ISTH) criteria is shown in Table 5. 

Moreover, thrombotic microangiopathy (TMA) in the context of hyperinflammatory conditions, such as AoSD, is another feared coagulation disorder. TMA causes small vessel thrombosis and could lead to strokes or multi-organ failure. Acute blurred vision may be an early symptom of ocular involvement in TMA [84].

## 8. Treatment Management

**Figure 1:**
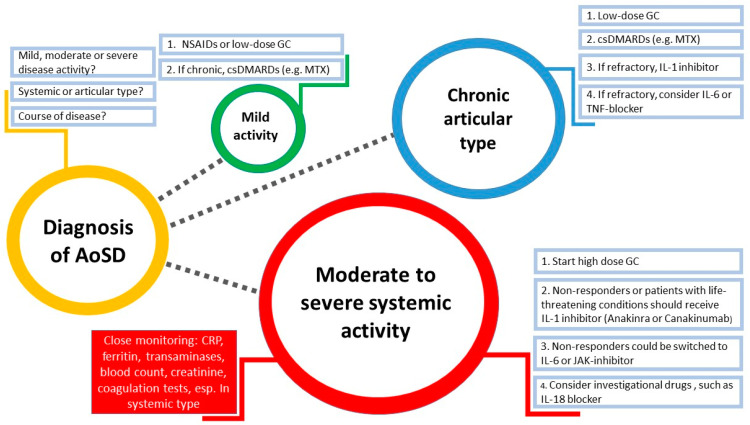
Suggested strategy for management of AoSD. Diameter of the circles represents the challenge in clinical practice. AoSD = Adult-onset Still’s disease, NSAIDs = Non-steroidal anti-inflammatory drugs, MTX = Methotrexate, csDMARDs = Conventional synthetic disease-modifying antirheumatic drugs, IL = Interleukin, TNF = Tumor necrosis factor, JAK = Janus kinase, GC = Glucocorticoids, CRP = C-reactive protein.

The establishment of a default management strategy for rare diseases such as AoSD is not easy (Figure 1). Steroids and NSAIDs are almost always the first-line treatment regimen in both clinical phenotypes; unfortunately, they have a poor overall response. To achieve satisfactory control of the disease, many physicians offer their patients disease modifying antirheumatic drugs (DMARDs) such as methotrexate, ciclosporin or azathioprine, although there is no robust evidence to support this practice [85]. However, the anticipated response rate in patients with the chronic articular phenotype of AoSD should be higher when the therapy protocol for rheumatoid arthritis is adopted [67]. 

Moreover, systematic reviews on AoSD are problematic because of the heterogeneity of clinical disease courses, the different organ manifestations and used treatment approaches. There is a great need for large multicentric prospective trials.

### 8.1. Anti-TNF Therapy

In contrast to numerous trials in the field of rheumatoid arthritis and spondyloarthritis, the efficacy of TNFα blockers in AoSD is controversial. They should probably only be prescribed for patients in the end stage of the articular type to inhibit erosion progression [86,87,88]. 

### 8.2. Anti-IL-1 Therapy

Evidence over the last twenty years has explained the central functional role of IL-1 in the pathogenesis of autoinflammatory conditions. Anakinra, a recombinant humanized IL-1 receptor antagonist, is the first choice for AoSD, yet patients with mainly articular phenotypes do not always benefit. Anakinra is licensed for subcutaneous use for systemic juvenile idiopathic arthritis, periodic fever syndromes, rheumatoid arthritis and AoSD (only by the European Medicines Agency) [12,89,90,91]. Rapid improvement in the systemic features of AoSD following anakinra administration was well demonstrated in a recent large observational retrospective multicentre study in 140 Italian patients [92]. 

However, the slower absorption of the subcutaneous route is a major disadvantage when facing a cytokine storm in patients with critical illness. This issue was addressed in a study with 46 patients with MAS, where 18 of them were treated with intravenous anakinra. Its pharmacokinetic and safety profile looks promising, yet the dosing scheme remains unclear. The authors concluded that intravenous anakinra could be used as a first-line treatment in MAS [69]. 

The other strategy for inhibiting IL-1 that has been intensively studied to date consists of a fully human antibody against IL-1β, canakinumab [93,94,95]. Canakinumab is currently licensed for AoSD, SoJIA, periodic fever syndromes and gout [96]. The CONSIDER study (Canakinumab for Treatment of Adult-Onset Still’s Disease to Achieve Reduction of Arthritic Manifestation), a phase II, randomized, double-blind, placebo-controlled, multicentre, investigator-initiated trial was terminated prematurely and did not reach the primary outcome (ΔDAS28 > 1.2). However, this trial demonstrated that in AoSD, treatment with canakinumab yielded improvement in several clinical aspects of the disease, while showing a favourable safety profile [97,98,99].

The efficacy and safety of another IL-inhibitor, rilonacept, was analyzed in a randomized, double-blind, placebo-controlled trial with seventy-one children with SoJIA. Rilonacept showed some benefit with an acceptable safety profile, although the primary end point was not met [100]. 

### 8.3. Anti-IL-6 Therapy

Tocilizumab, a humanized monoclonal antibody against the IL-6 receptor, showed promising results in the treatment of AoSD in a pilot study. Both the systemic features and the arthritic manifestations improved [101,102,103]. A 2018 meta-analysis investigated the benefits of tocilizumab in patients with AoSD and definitely showed signals of efficacy compared to conventional therapy regimes and was well acceptable in terms of safety [104]. The other IL-6 receptor antagonist, sarilumab, was reported to be effective as a steroid-sparing agent [105]. 

### 8.4. JAK Inhibitors

Contrary to anti-IL-1 and anti-IL-6 therapies, Janus kinase (JAK) inhibitors block a wide variety of proinflammatory cells and can therefore become a very promising treatment approach in heterogeneous disorders, such as AoSD. In a study with 14 patients with refractory AoSD, seven of them achieved complete remission under tofacitinib, while six responded partially. This trial also showed the steroid sparing effect of tofacitinib, especially in the articular phenotype [106]. Furthermore, a reported case of AoSD complicated by MAS describes remission with tofacitinib after failure of response to tocilizumab [107]. Another case report describes successful treatment of AoSD with tofacitinib in a HIV-positive female patient [108]. Baricitinib could also be an option, although current data are debatable [109].

### 8.5. Anti-IL-18 Therapy

Given the new insights into the pathogenic role of IL-18 in AoSD, this cytokine quickly became a drug target. Tadekinig alpha, a recombinant human IL-18 binding protein, demonstrated its potential effectiveness and acceptable safety profile in a phase 2 multicentred European study in 2018. The low number of participants (21) and the short period of treatment duration (12 weeks) could be considered limiting factors [110]. 

## 9. Still’s Disease in Children and Adults—Is There a Difference?

SoJIA was first described by Georg F. Still in 1897 as a novel disease entity differing from other forms of juvenile onset arthritis [111]. As AoSD and SoJIA share certain symptoms, it is worth investigating whether AoSD is a continuum of SoJIA in adult patients. 

A closer look at the age-dependent disease prevalence gives first indications that the two entities may be a continuum of one and the same entity. Unpublished data from German registries for pediatric and adult patients with rheumatic diseases yields a continuous decline of SoJIA prevalence by age with highest prevalence in age group 0–4 years. The prevalence in the 15–20 years old patients closely corresponds to the prevalence of young adults with AoSD, which also further declines by age. 

Even on closer inspection, SoJIA and AoSD show multiple other similarities. The differences described below may be based primarily on different research strategies as well as the inclusion of various patient cohorts and therefore do not contradict the thesis of a common disease continuum. Understanding the pathology and clinical manifestation of both entities should therefore be considered synergistically to identify age-dependent differences and define age-independent similarities.

Investigating drivers of paediatric diseases frequently focus on underlying genetic conditions. Therefore, multiple genome association studies have been performed to answer whether the fire ignites particularly easily in the presence of a certain genotype. First genetic studies on SoJIA were already published in 1976 and showed that SoJIA differs from other forms of JIA [112]. More recently a locus on chromosome 1 and loci within the HLA class II and III region on chromosome 6 have been associated with SoJIA [113]. Furthermore, HLA-DRB1*11 was found to be a major risk factor for SoJIA indicating the involvement of antigen-specific T cells [114]. In combination, these studies suggest a complex pathogenesis with multiple levels of genetic diversity. Furthermore, there is also emerging evidence for a rare familial monogenic form of SoJIA, which is associated with mutations in *LACC1* leading to a reduced autophagy flux in primary macrophages [115,116,117]. 

In SoJIA, a model assumes a biphasic disease course with an initial systemic phase dominated by fever, followed by an intermediate phase and finally a phase in which arthritis is in the foreground [118]. Especially during the early phase of SoJIA PAMPs and DAMPs, prominently S100A8/9 and SA10012 initiate a fever-syndrome with signs of autoinflammation [119,120]. In this respect, it was shown that leukocytes from SoJIA patients overreact to TLR4 and TLR 8 stimuli leading to a strongly increased IL-1 production by monocytes [121]. In a landmark paper, Pascual et al. described that sera derived from patients with SoJIA can induce–amongst others–the transcription of IL-1 in cells derived from healthy controls [122]. Additionally, unstimulated cells from patients with active SoJIA and AOSD express genes related to innate immunity including members of the IL-1 pathway [3,122]. This perspective is broadened by recent work, which demonstrate an association of high expression of certain transcription factors with early active SoJIA, indicating a role of B-cell activation and autoimmunity during that phase of disease [123].

Similar to AoSD it is suggested, that this first autoinflammatory phase is furthermore sustained by IL-18 and IL-6 [124]. IL-18 is part of the IL-1-family and induces the expression of Interferon-γ mainly by cytotoxic lymphocytes, which robustly express the IL-18 receptor. The naturally occurring antagonist IL-18 binding protein (IL-18BP) which is again induced by Interferon-γ controls the action of IL-18 [125]. 

Due to their central role in the differentiation of Th17 cells, the two cytokines IL-1β and IL-6 may be a key to understand the disease evolution of SoJIA [126,127]. Two recent studies analysing cells from patients with SoJIA give evidence that an IL-1-blockade prevents and/or reverses the differentiation of ɣ/δ T cells and regulatory T cells into a Th17 phenotype [128,129]. 

A central role of an impaired NK cell function in the perpetuation of SoJIA pathogenesis has been studied by analysing patient’s cells as well as applying a corresponding murine model [130,131,132]. 

Differently from adult patients, MAS is well known in paediatric patients. The transition from SoJIA to MAS (for classification criteria see Table 6) is sought to be initiated by the constant inflammatory trigger and often corresponds to a massive increase in IFNγ. Furthermore, as effective treatment of SoJIA using IL-1 and IL-6 blockade does not completely protect from MAS in these patients further mechanism must be involved in the development of MAS [78]. There is evidence, that a major driver for MAS is free IL-18 overcoming the inhibitory levels of IL-18-binding protein [125]. The close link of SoJIA to MAS is further demonstrated by whole-exome-sequencing showing overlaps between both diseases [133].

Clinical presentation can vary but most patients initially present very ill. The most common initial clinical features are fever, (most commonly a polyarticular) arthritis, and rash. Especially a fever pattern with one or two peaks on a daily basis, with rapid return to baseline is highly suggestive. The fever is classically accompanied by a discrete, salmon pink, erythematous macular rash. Furthermore, inflammatory affections of all organs can occur [135]. Emerging evidence for a lung disease, a rare but life-threatening complication in SoJIA patients comes from a multicentre retrospective study. The found pathology was mostly an alveolar proteinosis and lung disease was associated with macrophage dysfunction. Contrary to AoSD very young age was a predictor and despite a suggested macrophage dysfunction it is not associated with MAS in paediatric patients [74]. 

Similar to the AoSD classification criteria from Yamaguchi [57] and from Fautrel [43] the International League of Associations for Rheumatology (ILAR) defined criteria for the diagnosis of SoJIA (see Table 3) [58]. As the paediatric criteria require the presence of an arthritis a subset of SoJIA patients can have a severe delay in diagnosis as the systemic symptoms can proceed the arthritis by up to 10 years [135]. Retrospective testing of the Yamaguchi criteria in paediatric patients with suspected SoJIA with and without arthritis has yielded promising results especially for SoJIA patients with a delay in onset of arthritis [136]. Combining both sets of criteria might improve the time until diagnosis, especially in patients with a long time between systemic onset and beginning of arthritis [137]. A treatment window targeting the cytokine driven first phase of the disease might otherwise close [138].

**Figure 2:**
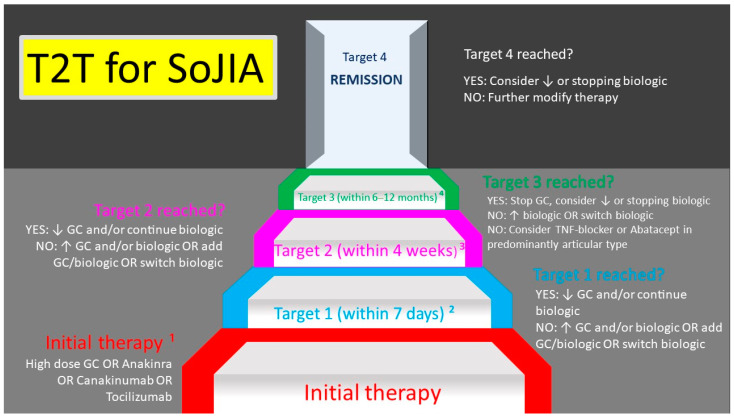
Treat-to-target consensus treatment strategy from the German Society for Pediatric Rheumatology for definitive SoJIA. In addition, non-steroidal anti-inflammatory drugs, intraarticular GC or Methotrexate may be used throughout.^1^ Maximal doses for glucocorticoids: intravenous methylprednisolone pulse therapy (20–30 mg/kg/day (max. 1000 mg/day) for 5 days or prednisolone equivalent 1–2 mg/kg/day (max. 80 mg/day). Maximal doses for biologics: Anakinra 8 mg/kg/day (max. 300 mg/day), Canakinumab max. 300 mg every 4 weeks, Tocilizumab (for body weight > 30 kg) 8 mg/kg (max. 800 mg) i.v. every 2 weeks and (for body weight < 30 kg) 12 mg/kg every 2 weeks.^2^ Treatment target 1-definition: resolution of fever or improvement of CRP by at least 50%. ^3^ Treatment target 2-definition: improvement of the physician global assessment by at least 50% AND reduction of the active joint count by at least 50% OR JADAS-10 score of maximally 5.4. ⁴ Treatment target 3-definition: clinically inactive disease without GC. T2T = Treat to target, SoJIA = Systemic-onset juvenile idiopathic arthritis, GC = Glucocorticoids, TNF = Tumor necrosis factor, JADAS = juvenile arthritis disease activity score (JADAS), “Biologic” refers to Anakinra, Canakinumab or Tocilizumab. ↓ = reduce drug dose. ↑ = increase drug dose.

Consensus-based treatment strategies exist from the German Society for Pediatric Rheumatology (GKJR) (Figure 2) [139] and the North American Childhood Arthritis and Rheumatology Research Alliance (CARRA) [140]. These are summarized as “treatment-to-target”. The main goal is achieving a clinical remission with the secondary goal of avoiding long-term glucocorticoids [139]. According to the CARRA and GKJR consensus statements methotrexate therapy is an option in articular diseases courses, either as sole long-term or adjunctive therapy. Besides glucocorticoids, IL-1 [141] and IL-6-receptor-targeting drugs are established cornerstones of modern therapeutical approaches [142]. The later ones have been proven successful in randomized trials [141,142,143]. Use of biologics is already suggested for initial treatment as monotherapy [139], whilst only results for initial treatment with anakinra have been published [144]. Furthermore, current data from the German National Pediatric Rheumatologic Database shows an increased usage of these biologicals as well as an improved initial response to treatment [145]. Other explanations for this effect could be an improved access to specialized care and a more rapid start of treatment. Furthermore, patient recruitment for a trial with the Janus-kinase bariticinib is active (ClinicalTrials.gov Identifier: NCT04088396). There is also an ongoing trial of tofacitinib in children with SoJIA (ClinicalTrials.gov Identifier: NCT03000439).

Although individual studies show certain differences between AOSD and SoJIA, a comparative analysis indicates that both findings most likely describe different ends of a common disease continuum.

## 10. Conclusions

AoSD is characterized by pathogenic involvement of both arms of the immune system. Despite extensive progress in understanding the pathophysiology and targeting the right cytokines, there are few large prospective cohort studies and randomized trials compared to other rare diseases, such as vasculitis. The new dichotomous classification of patients with AoSD into systemic and articular phenotypes may be a simple but very important step in designing and conducting future clinical trials. Furthermore, the development of activity score and treatment to target is required. These tasks should be addressed in cooperation between paediatric and adult rheumatologists. 

## Figures and Tables

**Table 1:** Differential diagnosis of AoSD [12].

Infections	Tuberculosis, toxoplasmosis, brucellosis, yersiniosis HIV, Epstein-Barr, cytomegalovirus, hepatitis, herpes, influenza, parvovirus B19, measles, rubella
Malignancies	Lymphoma, Castleman disease, myeloproliferative disorders, melanoma and colon, breast, lung, kidney and thyroid cancer In pediatrics also: leukemia
Systemic diseases	Systemic lupus erythematosus, idiopathic inflammatory myopathies, vasculitis, hereditary autoinflammatory syndromes, neutrophilic dermatosis, Sweet syndrome, reactive arthritis, sarcoidosis, Schnitzler syndrome, Kikuchi-Fujimoto disease In pediatrics also: other types of inflammatory arthritis

**Table 2:** Comparison of clinical features (%) of patients with AoSD and SoJIA.

	Di Benedetto P, Cipriani P, Iacono D, et al. (2020) [40]	Hu QY, Zeng T, Sun CY et al. (2019) [41]	Sfriso P, Priori R, Valesini G, et al. (2016) [35]	Gerfaud-Valentin M, Maucort-Boulch D, Hot A, et al. (2014) [42]	Fautrel B. et al. (2002) [43]	Tsai H. et al. (2012) [44]	Behrens E. D. et al. (2008) [45]
Case number	147	517	245	57	72	28	136
Nationality	Italy	China	Italy	France	France	Taiwan	United States
Female	39.5	72	47.3	53	nk	53.6	54
Average age at onset	45.2	37.7	38.8	36	35.2	8.7	5.7 Median 2
Fever ≥ 39 °C	100	91.3	92.6	95	84.7	100	98
Rash	74.8	79.9	67.7	77	70.8	67.9	81
Arthralgia/arthritis	88.4	73.1	93	95	88.8	89.3	88
Sore throat	56.5	60.5	62	53	52.7	nk	nk
Lymphadenopathy	54.4	51.1	60.4 *	60	44.4 *	46.4	31
Hepatomegaly	nk	6.6	41.7	21	nk	nk	~7
Splenomegaly	66.7	34.4	60.4 *	30	44.4 *	21.4 *	~5
Pericarditis	21.1	14.1	17.3	19	20.8	nk	10
Pleuritis	19.7	nk	nk	18	nk	7.1 *	nk
Myalgia	64.6	32.5	nk	44	nk	nk	nk
AoSD pneumonia	12.2	nk	nk	nk	nk	nk	nk
Abdominal pain	13.6	nk	nk	18	nk	nk	nk

nk = not known. * reported together as single variable.

**Table 3:** Classification criteria for AoSD and the revised definition of the International League of Associations for Rheumatology (ILAR) diagnostic criteria for SoJIA.

Criteria	1992 Yamaguchi [57]	2002 Fautrel [47]	2004 ILAR [58]
Major	Fever ≥39 °C lasting ≥1 week Arthralgia or arthritis ≥ 2 weeks Typical rash Leucocytosis ≥ 10,000/µL with ≥80% neutrophils	Spiking fever ≥39 °C Arthralgia Transient erythema ≥80% granulocytes Pharyngitis Glycosylated ferritin ≤20%	Arthritis in at least one joint Fever >2 weeks, daily for at least 3 days
Minor	Sore throat Lymphadenopathy Hepatomegaly or splenomegaly Abnormal liver function tests Negative rheumatoid factor and anti-nuclear antibodies	Maculopapular rash Leucocytes ≥10,000/µL	Evanescent erythematous rash Generalized lymph node enlargement Hepatomegaly Splenomegaly Serositis
Exclusion criteria	Infection, malignancy or other rheumatic disorders than mimic AoSD	None	Other forms of JIA must be excluded
Algorithm	Five criteria, at least two major ones AND no exclusion criteria	Four major criteria OR three majors with two minor ones	All major criteria AND at least one minor criteria
Sensitivity	96.2%	80.6%	Not applicable
Specificity	92.1%	98.5%	Not applicable

**Table 4:** HScore † ‡ for diagnosis of haemophagocytic lymphohistiocytosis [72].

Variable	Number of Points
Temperature	
<38.4 °C	0
38.4–39.4 °C	33
>39.4 °C	49
Organomegaly	
None	0
Hepatomegaly or splenomegaly	23
Hepatomegaly and splenomegaly	38
Cytopenia	
One lineage	0
Two lineages	24
Three lineages	34
Triglycerides (mmol/L)	
<1.5	0
1.5–4.0	44
>4.0	64
Fibrinogen (g/L)	
>2.5	0
≤2.5	30
Ferritin (ng/mL)	
<2000	0
2000–6000	35
>6000	50
Serum aspartate aminotransferase (IU/L)	
<30	0
≥30	19
Haemophagocytosis on bone marrow aspirate	
No	0
Yes	35
Known immunosuppression	
No	0
Yes	18

† The probability of having haemophagocytic syndrome ranges from <1% with an HScore of ≤90 to >99% with an HScore of ≥250. ‡ The HScore is freely available online (http://saintantoine.aphp.fr/score/ (accessed on 19 September 2020)).

**Table 5:** DIC-Score by ISTH [83].

Variables	Points
Platelet count (/µL)	
50,000–100,000	1
<50,000	2
Prolongation of PT (seconds)	
3–6	1
>6	2
Fibrinogen (mg/dL)	
<100	1
D-dimer (µg/mL)	
0.5–1	1
1–2	2
>2	3
If score ≥ 5: compatible with DIC. Repeat daily. If score < 5: suggestive of DIC. Repeat after 1–2 days.	

DIC = Disseminated intravascular coagulation, ISTH = International Society on Thrombosis and Haemostasis.

**Table 6:** Classification criteria for MAS in SoJIA (EULAR/ACR-approved [134]).

Major criteria	Febrile patient with (suspected) SoJIA Serum ferritin > 684 ng/mL
Minor criteria	Platelet count ≤ 181 × 109/L Aspartate aminotransferase > 48 U/L Triglycerides > 156 mg/dL Fibrinogen ≤ 360 mg/gL
Algorithm	Both major criteria with at least two minor criteria

## Abbreviations

AoSD	Adult-onset Still’s disease
SoJIA	Systemic-onset juvenile idiopathic arthritis
MAS	Macrophage activation syndrome
HLH	Haemophagocytic lymphohistiocytosis
PRRs	Pattern recognition receptors
NLR	Leucine-rich repeat containing family
PAMPs	Pathogen-associated molecular patterns
DAMPs	Damage-associated molecular patterns
IL	Interleukin
TNF	Tumor necrosis factor
GF	Glycosylated ferritin
DIC	Disseminated intravascular coagulation
TMA	Thrombotic microangiopathy
PET/CT	Positron Emission Tomography/Computer Tomography
ILAR	International League of Associations for Rheumatology
